# Temporal and spatial distribution of pectin, β(1–4)-galactan, xylan and lignin during differentiation of living fibres in young shoots of *Leucaena leucocephala* (Lam.) de wit

**DOI:** 10.1186/s12870-025-07793-z

**Published:** 2025-12-01

**Authors:** Pramod Sivan, K. S. Rajput, Karumanchi S. Rao

**Affiliations:** 1 Department of Chemistry, Division of Glycoscience, KTH Royal Institute of Technology, AlbaNova University Centre, Stockholm, 106 91 Sweden; 2 Department of Botany, The M.S. University of Baroda, Vadodara, 390 002 India; 3 Department of Biosciences, Sardar Patel University, Vallabh Vidyanagar, Gujarat 388 120 India

**Keywords:** Living fibres, Leucaena leucocephala, Cell wall polymers, Immunofluorescence, Transmission electron microscopy

## Abstract

**Background:**

Living xylem fibres play important physiological and mechanical functions in trees. Despite a significant role of secondary cell wall chemical composition in performing these functions, little information is available on the developmental changes in the cell wall of living fibres. In the present study, the distribution pattern of pectins, hemicelluloses, and lignin in the cell walls during the differentiation and maturation of living fibres in the secondary xylem of *Leucaena leucocephala* was examined by light and electron microscopy.

**Results:**

The expansion of primary walls during the early stage of fibre development was characterised by a change in the organisation of pectic polysaccharides in the middle lamellae region. The intercellular regions became filled with pectic polysaccharides following initiation of secondary wall deposition. Subsequently, lignification started at cell corners with the deposition of guaiacyl units that co-polymerise with syringyl moieties in the final stages of fibre development. The transmission electron microscopic analysis confirmed the disorganisation of pectic polysaccharides in the middle lamellae region during cell expansion and their inhomogeneous distribution in the cell corners following secondary wall deposition. Immunofluorescence microscopy revealed that β(1–4)-galactan are mainly incorporated in the middle lamellae region that undergoes disorganisation and reorganisation during and after cell expansion. Immunogold labelling experiments using JIM5, JIM7 and CCRCM1 antibodies revealed dynamic changes in the distribution pattern of homogalacturonan with different degrees of methylation and fucosylated xyloglucan during cell wall loosening and secondary wall maturation stages of xylem fibres. In mature fibres, LM10 labelling indicated that the less substituted xylans are distributed throughout the secondary wall, while labelling of highly substituted xylans with LM11 appeared more intense at the corner regions of the secondary wall compared to other regions. The KMnO_4_ staining revealed the relatively higher lignin distribution in xylem fibres in compound middle lamellae and S_3_ wall layers. The transition zone between S_1_ and S_2_ layers showed relatively high lignin distribution compared to the rest of the S_2_ wall layer. The ultrastructural studies demonstrated that the inhomogeneous distribution of lignin corresponds with that of pectins at the cell corners of fibres. The cell wall delignification resulted in a significant reduction of lignin at cell corners, compound middle lamellae and secondary wall layers of fibres.

**Conclusion:**

This study revealed the dynamic developmental changes in the structure of the cell wall during secondary wall development by assembly of cell wall polysaccharides and lignin during differentiation of living fibres in *L. leucocephala*. These insights are very important to understand the development and functional dynamics of wood fibres.

**Supplementary Information:**

The online version contains supplementary material available at 10.1186/s12870-025-07793-z.

## Introduction

 Plant fibres represent one of the most important renewable resources used as raw materials for many commercial purposes. From a functional perspective in planta, fibres are important in establishing plant architecture, as a source of mechanical support, in defence from herbivory and in some cases as elements with contractile properties resembling those of muscles [1, 2]. Wood is a major energy source for many in the world, and its energy content is mainly correlated with the lignified fibres [2]. The length and chemistry of xylem fibres are considered critical quality parameters for the paper industry [3]. To meet the ever-increasing demand of wood fibres for the paper industry and to reduce the pressure on the natural forests, tree species producing wood with higher quality fibres have to be planted on less land. Moreover, with their extreme length and thick cell walls, fibres form an attractive model for the study of plant cell elongation and cell wall formation.

In the majority of plants, xylem fibres become dead at the final stage of cell maturation and function for mechanical support, while certain species are characterised by living fibres. The developmental changes in fibres during xylogenesis mainly involve structural and chemical modifications of the cell wall, resulting in the formation of a thick secondary wall (SW). The major structural changes include expansion, elongation, and wall thickening, while the chemical changes involve deposition of polysaccharides, i.e., cellulose (major constituent), hemicelluloses and pectin (matrix polysaccharides) and incorporation of phenylpropanoid derivative, lignin, into the matrix. The hemicellulose composition of xylem fibres in hardwood is composed mainly of glucuronoxylan and a small amount of glucomannan [4]. Lignin is a complex and irregular poly-phenylpropanoid heteropolymer that enhances the structural integrity of the cell wall [5]. It imparts rigidity to the cell walls [6] and is responsible for the decay resistance of wood [7]. On the other hand, as a result of the chemical and structural properties tailored for these functional roles, the presence of lignin has many negative impacts during postharvest deconstruction of lignocellulosic biomass for commercial applications. Lignin is an undesirable component during the manufacture of paper and pulp, and the chemical degradation of lignin during this process is neither an economically nor environmentally friendly process. Therefore, lignin quantity and its monomeric composition, which vary from species to species, have been considered as one of the major characteristics while selecting suitable plants for the paper and pulp industry. The living fibres are different in terms of cellular structure and function as they possess intact cytoplasm and starch reserves in plastids. Although differentiation of dead xylem fibres has been studied extensively, there is little information available on the cell wall development in living fibres.

*Leucaena leucocephala* is an evergreen tree species that belongs to the family Fabaceae, growing in the tropics and subtropics. It became one of the most versatile fast-growing commercially important trees for the paper and pulp industry in India, contributing nearly a quarter of the total raw material [8]. The fast-growing nature, adaptability to a variety of soils and climatic conditions and chemical composition, particularly high cellulose and low lignin contents, made its wood suitable for the manufacture of paper and pulp. *Leucaena* produces fibres in bark and wood, where the latter is of commercial importance. The secondary xylem consists mainly of fibres, which account for more than half of the wood elements. The structure and chemistry of the secondary cell wall of fibres determine the functional diversity and application of wood-based products. Looking into the economic viability of *L. leucocephala*, many studies have been carried out on the chemical composition [9, 10] and chemical modification of lignin through genetic engineering [11–13]. The *L. leucocephala* is characterised by the occurrence of living fibres in the xylem [14]. To the best of our knowledge, there is no detailed study on the structure and distribution pattern of cell wall polymers during the differentiation of living fibres in plants. Information on the distribution of individual cell wall polymers during secondary cell wall development in xylem fibres is essential for understanding the biological significance as well as the mechanical behaviour of wood, which provides opportunities for improving technologies for chemical wood processing. Since the cell wall chemistry is directly related to the developmental sequences during cell wall development, the purpose of the present study is to describe the spatial and distribution pattern of pectin, xylan and lignin during differentiation of living fibre in the secondary xylem of young shoots of *Leucaena leucocephala*.

## Materials and methods

### Plant material

For histological and histochemical studies, samples were collected from the 6-8th internode of actively growing shoots of one-year-old *Leucaena leucocephala* (Lam.) De Wit plants growing in the Botanical Garden of the Department of Biosciences, Sardar Patel University, Vallabh Vidyanagar, Gujarat, India. These plants were maintained for research purposes and samples were collected with permission from the administrative authority of the Botanical Garden.

### Delignification with sodium chlorite and LR white embedding

Delignification was performed according to the procedure described by Kim et al. [15]. Longitudinal sections of 500–600 μm thick were prepared from wood blocks using a sliding microtome and then delignified in 8% NaClO_2_ (in 1.5% acetic acid) at 40 °C for 48 h. Then the sections were washed several times with distilled water, followed by buffer wash (cacodylate buffer, pH 7.2). After washing in buffer, samples were dehydrated in a graded series of ethanol (30–95%, 15 min each, pure ethanol × 3, each for 20 min), infiltrated with a mixture of LR white and ethanol (V/V) 1:3, 1:1,3:1 and pure resin (4 days in each solution). For better infiltration, samples were kept under vacuum during the day and left on the rotator overnight. Embedding was done in fresh LR white using gelatin capsules, and capped capsules were left in the oven at 60 °C for polymerisation for two days.

### General histology

Semithin sections (1–2 μm) were taken from LR white embedded blocks with a glass knife using a Reichert OM U3 ultramicrotome. Sections were stained with 0.05% aqueous toluidine blue O with 1% borax and mounted in DPX. Stained sections were photographed using an Olympus BX 50 microscope attached with a DP 71 Digital Camera (Germany).

### Histochemical staining

For the histochemical localisation of pectins, 2–4 μm thick sections taken from LR white embedded blocks were stained with 0.02% aqueous ruthenium red for 5 min, washed with distilled water and mounted in DPX [16]. Localisation of cellulose and lignin was carried out using free-hand sections taken from the 6th internodes of young shoots. Lignin was localised in the xylem elements using the Wiesner reaction by pouring a few drops of 1% phloroglucinol in ethanol solution on the sections mounted on a glass slide, followed by adding a drop of 10% HCL [17]. Maüle reaction [18] was used to localise the abundance of syringyl lignin moieties in the cell wall. Sections were immersed in 1% KMnO_4_ for 5 min, washed in distilled water, immersed in 3% HCl for 1 min, washed in distilled water, added a few drops of ammonium hydroxide solution and covered with a glass slide. The sections were immediately observed and photographed using a Carl Zeiss Microscope (Axioplan, Germany) attached with a CCD camera.

### Fluorescence microscopy

For fluorescence microscopy, lignin autofluorescence was measured at green excitation (λ excitation at 510 nm and emission at 560 nm), and images were taken using a *Leica* DM 2000 epifluorescence microscope attached with a Canon DC 150 Digital Camera (Germany).

### Immunofluorescence microscopy

Stem samples after trimming to 2 × 5 mm size pieces were fixed in a mixture of 0.1% glutaraldehyde and 4% paraformaldehyde in 50mM sodium cacodylate buffer for 4 h at room temperature. After washing in buffer, the tissues were dehydrated in a graded series of ethanol (30–95%, 15 min each, pure ethanol × 3, each for 20 min). Samples were then embedded in LR white resin using the same method as described above.

Immunolabeling was carried out according to the method described by Kim et al. [19]. 1 μm-thick transverse sections were prepared from LR white embedded blocks with a diamond knife. Sections were mounted on formvar-coated slides and treated with 50 mM glycine/phosphate-buffered saline (PBS) solution for 15 min. Sections were washed with PBS buffer and were suspended in blocking buffer (PBS) containing 3% Skim milk for 30 min at room temperature. Sections were incubated in monoclonal antibodies (Plant Probes, UK; 1:100 dilution in PBS buffer) specific for β(1–4)-galactan (LM5) and Xylans (LM10, LM11) for 2 days at 4 °C. After washing in three changes of PBS buffer for 10 min each, sections were incubated with anti-mouse IgG Alexa Fluor 568 (Invitrogen, USA; 1:1000 dilution in PBS buffer) for 2 h at 35 °C. After washing in PBS buffer for 3 times (10 min each) and mounted in Fluoroshield (Sigma, Germany) on a clean glass slide, without toluidine blue O staining, sections were examined under a fluorescence microscope (Ziess, Axio Observer Z1, Germany) fitted with a filter combination of 570 nm exciter and a 603 nm emitter. Control sections were incubated without primary antibody treatment.

### Immunogold labelling

Ultrathin sections of 90 nm thickness mounted on nickel grids were used for suspension in buffer ‘A’ (composition: Tris-buffered saline containing 1% bovine serum albumin and 0.1% NaN3, pH 8.2) for 30 min at room temperature, the grids were incubated with LM5, JIM5, JIM7, CCRCM1, LM10 or LM11 antibodies (1:20 dilution in buffer A) for 2 days at 4˚C. The labelling method was the same for CCRCM1, JIM5 and JIM7, except the grids were incubated with goat anti-mouse secondary antibody labelled with 5 nm colloidal gold particle (BB International, UK). After three washes with buffer A for 15 min each, the grids were incubated with goat anti-rat secondary antibody labelled with 10-nm colloidal gold particles (BB International, UK) for 2 h at room temperature. For the control, some sections were also incubated only with the secondary antibody. Finally, the grids were washed in six changes of buffer ‘A’ for 15 min each, followed by washing with distilled water. Ultrathin sections were post-stained with uranyl acetate for 30 min, washed in three changes of distilled water for 10 min each.

### Transmission electron microscopy (TEM)

For the lignin localisation, samples were cut into 2 × 5 mm sized pieces and fixed in a mixture of 0.1% glutaraldehyde and 4% paraformaldehyde in 50 mM sodium cacodylate buffer for 4 h at room temperature and then embedded in LR white resin according to the procedure described above. For pectin detection, small pieces of differentiating xylem tissue samples were fixed for 4 h in a mixture of 0.5% ruthenium red (w/v) and 3% glutaraldehyde (GA) (V/V) in 50 mM cacodylate buffer (pH 7.2). Another set of samples were fixed by the modified micro-oven method [20] using Karnovsky’s fixative (combination of 8% glutaraldehyde and 8% paraformaldehyde in cacodylate buffer) for better preservation of cytoplasm in living fibres. Glass vials with samples were kept between two 500 ml beakers filled with water on the opposing corners of microwave oven for 30–35 s. This step was repeated 4 times with an interval of 5 min. The fixative solution was changed after each interval. After micro-oven treatment, samples were kept in a vacuum for 6 h on ice bags and the fixative was changed at 1 h intervals. Samples were then washed in the same buffer and post-fixed in 1% osmium tetroxide (w/v). After the routine dehydration and infiltration, the samples were embedded in Spurr’s resin [21]. Ultrathin sections of 60–80 nm thickness were taken using a diamond knife and collected on nickel grids. Sections were stained with 0.1% KMnO_4_ in citrate buffer for 45 min at room temperature for lignin [22]. Pectins were localised by staining ultrathin sections with 1% ruthenium red for 8 h [23]. For cell wall polysaccharides, sections taken on gold grids were subjected to periodic acid- thiocarbohydrazide-silver proteinate (PATAg) staining [24]. Samples fixed using the microwave oven method were subjected to uranyl acetate-lead citrate staining. Ultrathin sections after immunolabeling as well as other contrasting methods were observed under TEM (FEI, Tecnai G^2^, Philips, Morgagni M268 and JEOL JEM-1400) at an acceleration voltage of 80 kV. Micrographs were taken using a CCD camera (Mega View III, Olympus Soft Imaging Solutions, USA).

## Results

### Structural and histochemical changes in pectin and xylan in differentiating fibres

**Fig. 1:**
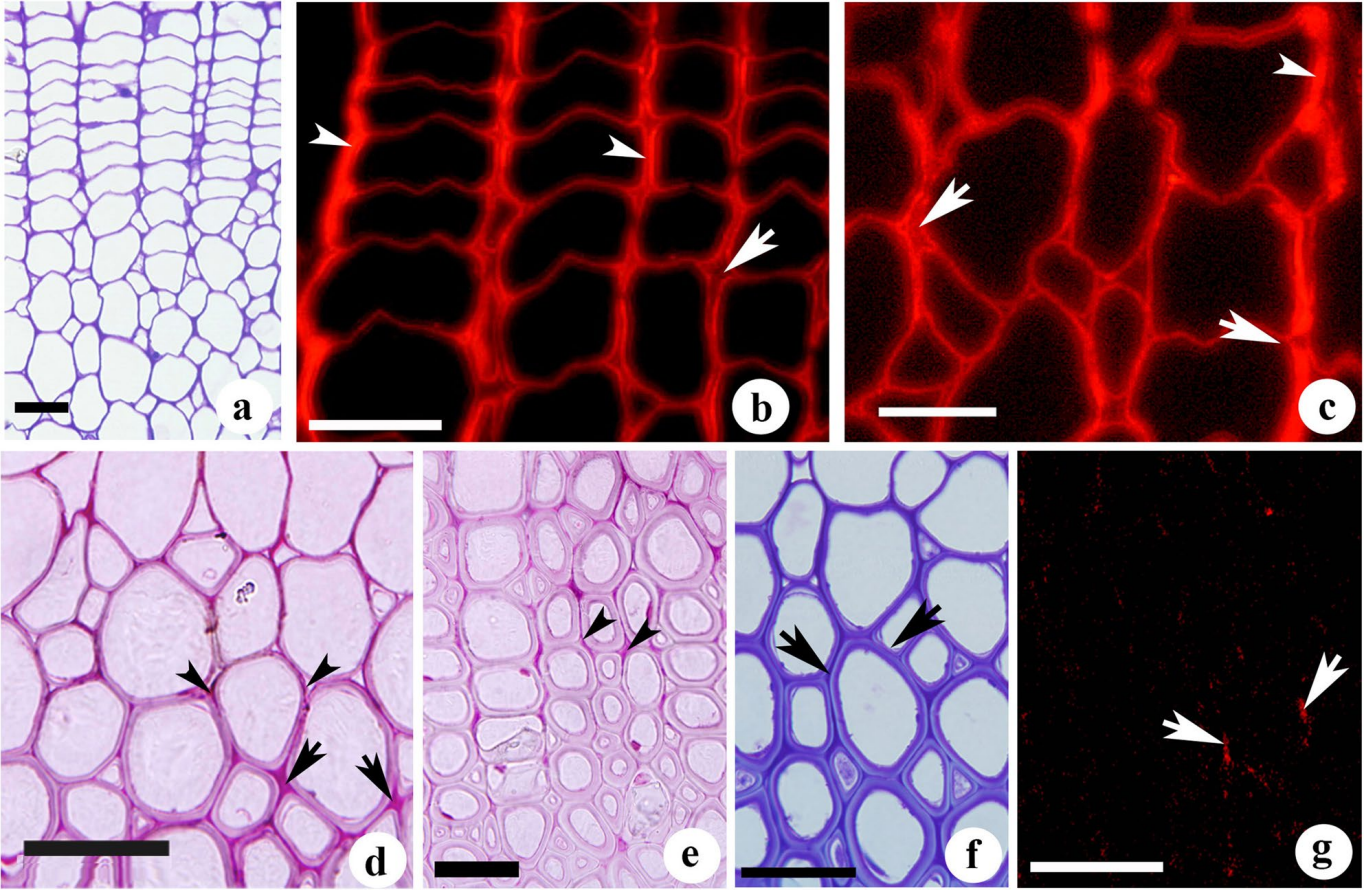
(a-g): Transverse sections stained with toluidine blue O (a, f), ruthenium red (d,e) and immunofluorescence (b,c,g) detection of β(1-4)-galactan (LM5) in the differentiating xylem of *Leucaena leucocephala*. (**a**) Cambial zone showing radial tiers of cambial cells and centripetal xylem derivatives undergoing expansion growth. (**b**) Detection of β(1-4)-galactan shown by strong labelling from the radial wall of cambial cells (arrowheads). Arrows indicate the absence of labelling from cell corners and ML regions following loosening of wall materials in the differentiating elements. (**c**) Primary wall (arrowhead) region of fibres showing LM5 labelling after completion of cell expansion. Arrows indicate the absence of fluorescence from the intercellular spaces. (**d**) Fibre walls undergoing secondary wall deposition showing intercellular spaces at the early stage (arrowheads). Arrows indicate intercellular spaces filled with pectins in the late stage. (**e**) Mature fibres showing gradual disappearance of pectic polysaccharides at cell corners (arrow heads) and middle lamellae region. (**f**) Xylem fibres undergoing secondary wall deposition. Arrows indicate the disappearance of intercellular spaces at the late stage of secondary wall deposition. (**g**) Fibres at the late stage of secondary wall deposition indicated in the ‘f’ showing labelling of β(1-4)-galactan from the cell corners and compound middle lamellae of fibres (arrows). Scale bar 25 µm

**Fig. 2:**
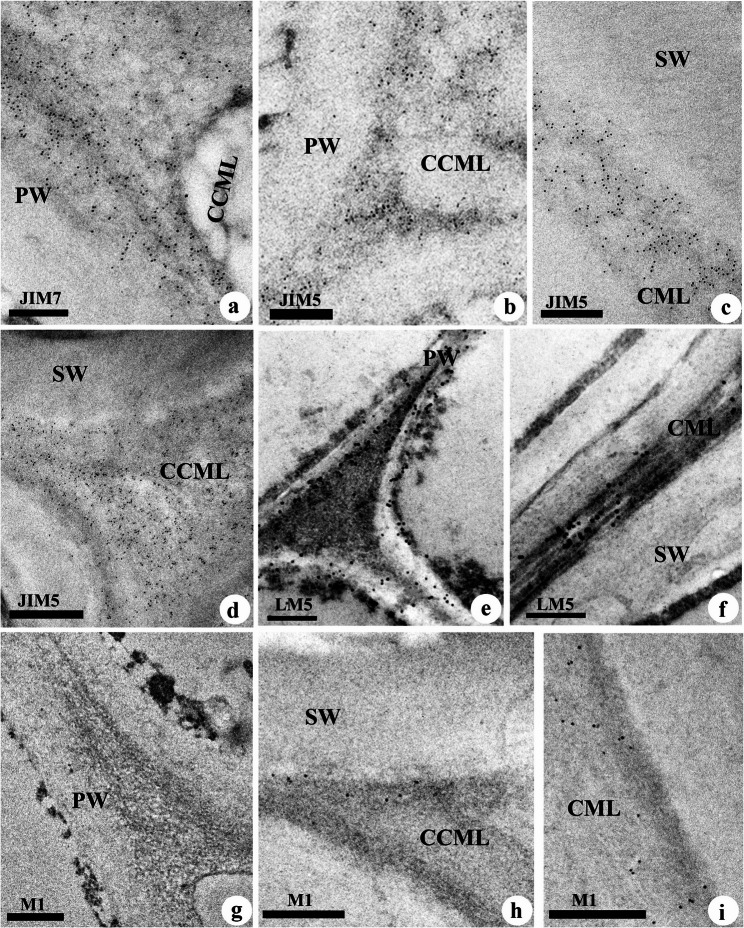
(a-i): Immunolocalisation of high methyl esterified homogalacturonan with JIM7 (**a**) low methyl esterified homogalacturonan with JIM5 (**b-d**), β(1-4)-galactan with LM5 (**e-f**) and fucosylated xyloglucan with CCRCM1 designated as M1 in the figure (g-i) in the differentiating fibre cell wall of *Leucaena leucocephala. *Note the strong labelling of JIM7 (**a**) while weak labelling for JIM5 (**b**) in the cell corner and middle lamellae region of fibres in the expansion zone. (**c,d**) Strong JIM5 labelling in the compound middle lamella (**c**) and cell corner (**d**) region of mature fibres. (**e**) fibres in the expansion zone showing strong LM5 labelling in the primary wall and middle lamella. (**f**) fibres with secondary wall (SW) showing LM5 labelling limited to the compound middle lamellae region. (**g**) weak M1 labelling in the fibre cell in the expansion zone. (**h-i**) Mature fibres showing M1 labelling in the cell corner middle lamellae (CCML) and compound middle lamellae (CML) regions. PW= Primary wall, ML= Middle lamella, CML= Compound middle lamella, SW= Secondary wall, CCML= Cell corner middle lamella. Scale bar= 250 nm

Transverse sections stained in toluidine blue showed cambial zone cells with thick radial walls, which gradually loosen during cell expansion (Fig. 1a). The labelling with LM5 revealed relatively high fluorescence intensity in the cell walls of the cambial zone and expansion zone, while no labelling was detected in the mature xylem. The cambial initials were demarcated by the high intensity labeling of radial walls (Fig. 1b). Loosening of wall materials in cambial derivatives was marked by the absence of labeling which initiated at cell corners and further spreads to radial walls (Fig. 1c). This indicates that β(1–4)-galactan is largely disorganized at the middle lamellae region during cell expansion and its disappearance along with other pectins could be an early indicator of cell differentiation from cambial cells. Ruthenium red staining revealed that the intercellular spaces formed after disappearance of pectic network was more prominent in the cells undergoing expansion growth, while those cells completed the expansion and undergoing secondary wall deposition showed reappearance of pectins as evident from the intense staining at cell corners and middle lamellae (ML) region (Fig. 1d). During the course of secondary wall deposition, pectin network was found distributed in the cell corner middle lamellae (CCML) and compound middle lamellae (CML) regions of fibres (Fig. 1e). Fibres showed occurrence of intercellular spaces till the late stage of secondary wall deposition (Fig. 1f). Immunolabelling with LM5 also showed strong labeling of β(1–4)-galactan in the compound middle lamella region of fibre undergoing secondary wall deposition (Figs. 1g and 2f). After the completion of secondary wall deposition, ruthenium red staining intensity for pectins became less intense and subsequently disappeared in mature elements (Fig. 1e). However, immunogold localisation of homogalacturonan (HG) revealed a heterogeneous distribution of high methyl esterified HG (JIM7) and low methyl esterified HG (JIM5) during different developmental stages of fibre development. JIM7 labelling (Fig. 2a) was relatively stronger than JIM5 (Fig. 2b) in the loosely organised cell corner region, suggesting enrichment of high methyl esterified HG during the cell wall loosening stage. On the other hand, dense distribution of gold particles for JIM5 labelling was noticed in the cell corner and middle lamellae region of fibres at advanced stages of secondary wall development (Fig. 2c, d), suggesting cross-linking of low methyl esterified pectins in the cell corner middle lamellae during fibre maturation. Immunolabeling approach also indicates the limitation of pectin staining in mature wood fibres with ruthenium red, possibly due to the presence of lignin.

Immunogold labelling of fucosylated xyloglucan with CCRCM1 (M1) suggested weak labelling in the fibre initials undergoing cell wall loosening (Fig. 2g). When the secondary wall formation advances with reorganisation of the cell corner region, M1 labelling was noticed in the cell corners and compound middle lamellae region (Fig. 2h, i). This heterogeneity in M1 labelling during fibre developmental stages suggests dynamic changes in the distribution of fucosylated XG occur during cell expansion stages.

**Fig. 3:**
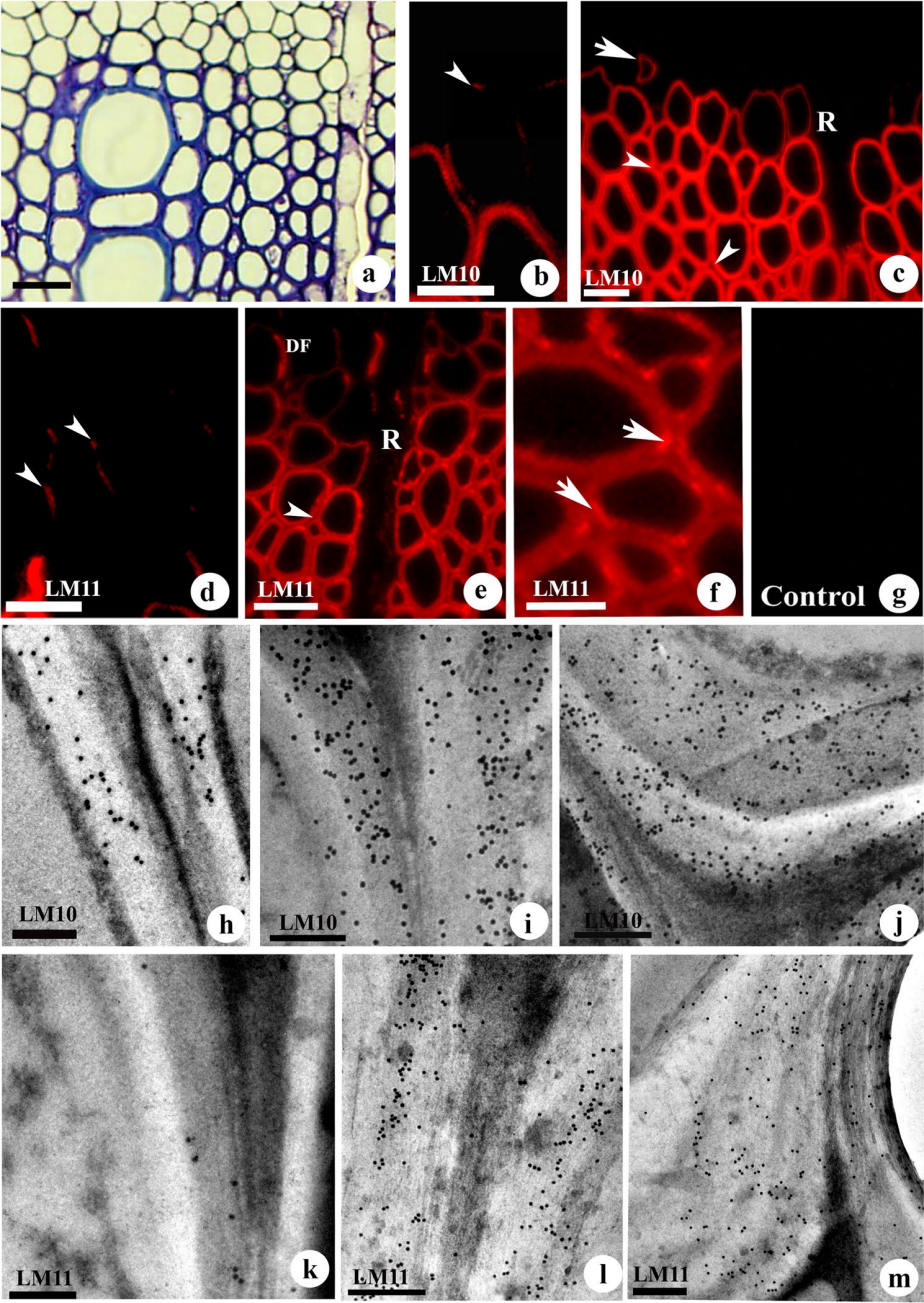
(a-e): Toluidine blue O staining (**a**) and immunofluorescence (**b-g**) and immunogold (**h-m**) localisation of low substituted xylans (ls ACGXs) with LM10 and highly substituted xylans (hs ACGXs) with LM11 in the developing fibre cell wall of *Leucaena leucocephala.* (**a**) Differentiating xylem showing different stages of secondary wall deposition and lignification of the fibre wall during xylem maturation. Note the ray cell with a primary wall, while the adjacent axial elements remain with a secondary wall. (**b**) Differentiating fibres showing detection of LM10 labelling initially at corners of secondary wall (arrowhead). (**c**) Detection of xylan (LM10) showing labelling from the tip region of fibres undergoing secondary wall deposition (arrow). Arrowheads indicate the weak labelling compound middle lamella of fibres. Note the absence of labelling in ray cell walls (R). (**d**). Early detection of LM11 labelling from the cell corner region of secondary wall of differentiating fibres (arrowheads). (**e**) Fibres showing weak labelling with LM11 at the CML region (arrowhead). Note the progression of xylan labelling in the walls of differentiating fibres (DF), while weak labelling from the ray (R) cells. (**f**) Mature fibres showing intense labelling for highly substituted xylans at cell corner regions (arrows). (**g**) Control tissue omitted with primary antibody showing absence of fluorescence labelling. (**h-m**) LM10 and LM11 labelling during the early stage of secondary wall formation (**h, k**) and after maturation of xylem fibres (i, j, l, m). Note uniform distribution of gold particles throughout secondary wall (SW) for LM10 labelling (**j**), while relatively high density of gold labelling with LM11 labelling in the corner region of secondary wall of mature fibres (m). Scale Bar: a-g = 25 μm, h-m = 250 nm

The variation in cell wall thickness in differentiating fibres contrasted with toluidine blue staining (Fig. 3a). Immunofluorescence detection of xylan with LM10 revealed the labelling of the tip region of fibres, while ray cells close to the differentiating zone showed no labelling (Fig. 3b, c), suggesting variation in timing of secondary wall deposition between elements of the axial and radial system. For both LM10 and LM11 labelling, differentiating fibres showed signals initially from cell corners of secondary wall (Fig. 3b, d) and intensity became stronger with advancement of SW development (Fig. 3c, e, h-i). Weak labelling was noticed for LM10 and LM11 at the compound middle lamella and intercellular spaces (Fig. 3b, e). Weak or no labelling was evident in the ray cells that remain with primary walls (Fig. 3c, e). An increase in the intensity of fluorescence labelling was noticed in the secondary wall region that bends at cell corners (Fig. 3f, m), suggesting incorporation of more highly substituted xylans in this region.

### Pattern and heterogeneity of lignification

**Fig. 4:**
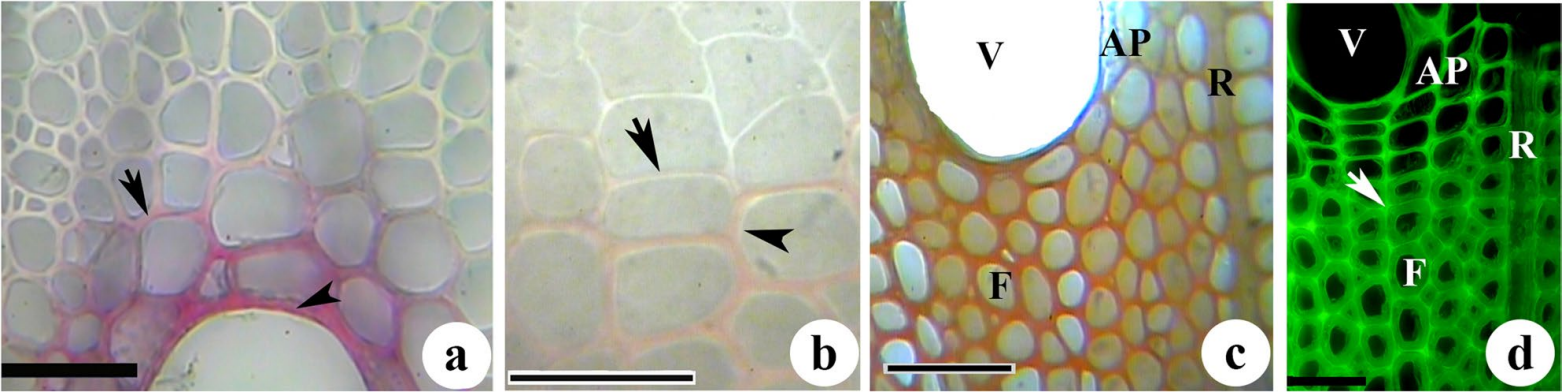
(a-d): Bright field (**a-c**) and fluorescence micrographs (**d**) from the transverse sections of the stem of *Leucaena leucocephala* showing lignification pattern during fibre development. (**a**) Intense lignification in the vessel (arrowhead) and associated parenchyma (Weisner reaction) compared to fibre (arrow). (**b**) Fibres showing deposition of G lignin units into compound middle lamellae region during early stage (arrow) and incorporation of more S lignin units (arrowhead) during late stage of lignification (Maüle reaction). (**c**) Fibre (**F**) walls showing a higher distribution of S lignin units. Note the presence of more G lignin units in the walls of the vessel (V), axial parenchyma (AP) and ray parenchyma (R) (Maüle reaction). (**d**) Relatively high autofluorescence from the CML region (arrow) of fibres indicates more condensed lignin units. Note the more autofluorescence from the vessel and associated parenchyma. Scale Bar = 50 μm

Lignin deposition first appeared in the walls of vessels, followed by paratracheal parenchyma cells (Fig. S_1_) and then ray parenchyma and fibres. The Weisner reaction of the secondary xylem revealed intense red color from the vessel walls while fibres showed pinkish red color indicating variation in relative deposition of lignin in cell walls of different xylem elements (Fig. 4a). Maüle reaction showed that fibre walls with brownish color during the initial stages of lignin deposition while they became reddish brown at mature stage (Fig. 4b). On the contrary, comparatively thick-walled vessels showed brownish red colour indicating guaiacyl-syringyl (GS) type lignin with more amount of guaiacyl (G) units (Fig. 4c). Maüle reaction of mature xylem elements revealed that fibre walls remain rich in syringyl (S) lignin while vessels, rays and axial parenchyma showed brownish color indicating incorporation of more amount of G units in their walls (Fig. 4c). Epi-fluorescence microscopy also revealed high autofluorescence from cell corners and compound middle lamella of fibres (Fig. 4d) which represents the area of early lignification (Fig. 4d). Relatively uniform pattern of higher fluorescence was observed (Fig. 4d) throughout the lignified cell wall of vessels and associated parenchyma than that of fibres.

### Ultrastructural changes during fibre differentiation

**Fig. 5:**
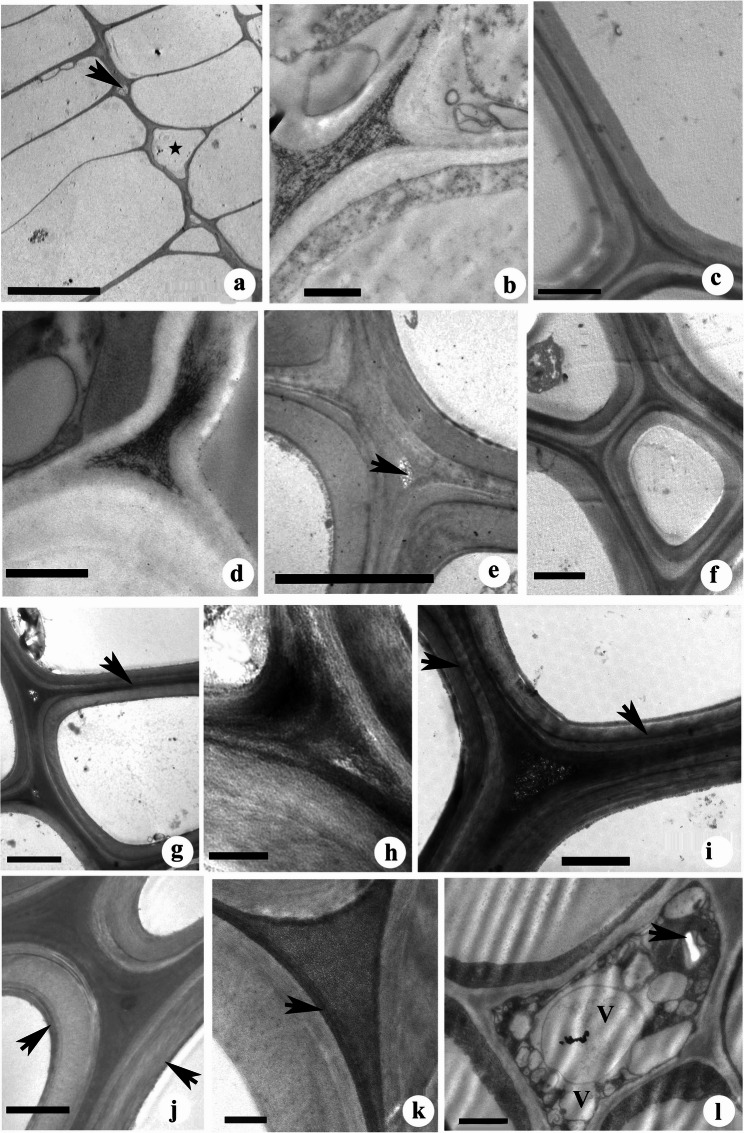
(a-l): Transmission electron micrographs from transverse sections of *Leucaena leucocephala* stained with PATAg (a, e,f) and ruthenium red (b-d, g&h), KMnO_4_ (g-k) and uranyl acetate-lead citrate (l) showing changes in cell wall polysaccharides during fibre differentiation. (**a**) Xylem derivatives near the cambium showing loosening of radial walls (arrow). Asterisk indicates a fibre tip showing a loosely organised cell wall. (**b**) Fibres in the expansion zone showing loosely organised pectic polysaccharides at the cell corner middle lamellae region. (**c**) Secondary wall deposition in the fibre. Note the relatively thin S_1_ layer in the outer wall region and the progressing deposition of the thick S_2_ wall layer. (**d**) Fibre undergoing secondary wall deposition showing pectic polysaccharides at the cell corners. (**e**) Fibres during the final stages of secondary wall deposition showing loosely organised CCML region with electron dense pectic polysaccharides (arrow). (**f**) Mature fibres after completion of secondary wall deposition showing different wall layers. (**g**) Fibres with a complete S_2_ wall layer showing intensely lignified CML (arrow) and progressive lignin distribution towards inner wall layers. (**h**) The CCML of the fibre shows inhomogeneous lignin distribution. (**i**) Fibre secondary wall showing more lignin distribution in CML and transition zone between S_1_ and S_2_ wall layers (arrows). Note the electron translucent areas in the cell corner region, indicating inhomogeneous lignin distributions. (**j**) Mature fibre after completion of secondary wall deposition and lignification showing relatively more lignin distribution in the innermost S_3_ wall layer (arrows). (**k**) The reticular structure with electron translucent regions (arrows) in the cell corner indicates the removal of lignin from the fibre. Note the electron-dense primary wall and less lignin distribution in the outer S_2_ layer. (**l**) A differentiating fibre showing intact cytoplasm in the lumen. Note the starch-filled plastids (arrow) and vacuoles (V). Scale bar: a, i, l = 1 μm; c,f, j = 2 μm; b,d, h, k = 0.5 μm

**Fig. 6:**
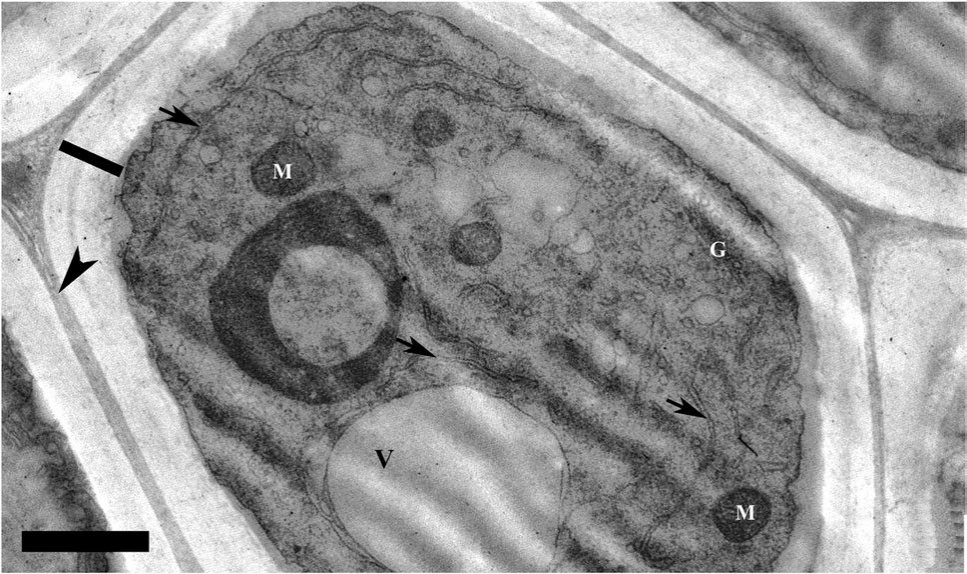
Cytoplasm in a mature, living fibre showing vacuoles (V), Golgi (G) and mitochondria (M), endoplasmic reticulum (arrows). The compound middle lamella and thick secondary wall are indicated by arrowhead and thick bar, respectively. Bar = 1 µm

The PATAg staining revealed loose organisation of the cell corner middle lamella in the xylem derivatives near the cambial zone, indicating cell wall expansion (Fig. 5a). Intrusive growth of fibre initials was noticed among the xylem derivatives in the expansion zone (Fig. 5a). The cell walls of differentiating xylem elements contrasted with ruthenium red showed inhomogeneous distribution of pectins. Pectic polysaccharides are mainly concentrated in the cell corner region of fibre derivatives adjacent to the cambial zone. The fibres adjacent to rays showed a large electron translucent region in the cell corner region, whereas the rest of the middle lamella was relatively densely stained (Fig. S2). Large electron translucent regions were found in the cell corner region of fibre cells undergoing expansion (Fig. 5b). The cell wall thickening begins with the deposition of S_1_ layer adjacent to primary wall followed by deposition of thick inner S_2_ layer (Fig. 5c). Ruthenium red staining revealed intact network of pectic polysaccharides in the cell corners of fibres undergoing secondary wall deposition (Fig. 5d). The maturation of fibres was marked by the deposition of a thin S_3_ layer (Fig. 5e). The ruthenium red staining also revealed the electron translucent regions in the cell corners of premature fibres with late stages of secondary wall deposition (Fig. 5e) which became electron dense after completion of secondary wall deposition and fibre maturation (Fig. 5f). The KMnO_4_ staining revealed the micro-distribution pattern of lignin in the fibre walls. The lignification of fibre walls begins at the cell corner region and spreads to the compound middle lamellae. After the deposition of the S_2_ wall layer, compound middle lamellae showed intense lignin distribution (Fig. 5g). The close examination of cell corners showed several electron translucent regions, indicating inhomogeneous lignin distribution in the cell corner area of fibres (Fig. 5h). The secondary wall also showed a distinct change in lignin distribution pattern. The transition zone between S_1_ and S_2_ layers was marked by relatively high lignin distribution (Fig. 5i). The fibres after completion of S_2_ layer deposition also showed electron translucent areas at cell corners (Fig. 5i). The inner S_2_ layer showed less lignin concentration whereas the thin layer of S_3_ layer was characterised by more lignin distribution (Fig. 5j). The maturation of fibres was also marked by the electron dense cell corner regions indicating lignin deposition. The KMnO_4_ staining of delignified cell walls showed significant removal of lignin from cell corner of fibres which was evident from the occurrence of several electron translucent spots in the cell corner middle lamellae region giving a reticulate structure while primary walls remain contrasted (Fig. 5k). Similar electron translucent areas were also found in the compound middle lamellae region (Fig. 5k). The lignin distributed in the outer S_2_ layers was mostly removed (Fig. 5k). The TEM analysis of differentiating xylem stained with uranyl acetate-lead citrate revealed the living fibres with intact cytoplasm during both early and mature stage of their development (Figs. 5l and 6). Starch-containing plastids were found in the cytoplasm of differentiating fibres (Fig. 5l). Dense cytoplasmic contents with vacuoles, mitochondria, endoplasmic reticulum and Golgi with vesicles were noticed in the fibres with secondary wall (Fig. 6).

## Discussion

The histochemical studies on cell wall differentiation in living fibres of *Leucaena leucocephala* young shoots reveal that major changes occur in the pectic polysaccharides during cell expansion and secondary wall deposition. Many of the observed developmental changes have been similar to the changes reported in dead fibres. Light and electron microscopic studies have indicated pectin-rich cell walls before cell expansion followed by disorganisation and disappearance of pectin during cell expansion. The disorganisation of the pectin network in the cell corner middle lamella (CCML) region results in the formation of intercellular spaces among fibres in the expansion zone. The stiffening and loosening of cambial cell walls have been attributed to Ca^2+^ cross-linking of acidic pectins (mainly homogalacturonan) and their deacidification through the methylation of carboxyl groups [25]. In the present study, the JIM5 and JIM7 labelling, which recognise low and high methyl esterified homogalacturonan [26], suggest the richness of JIM7 and weak JIM5 labelling in the cell corner middle lamellae undergoing loosening, indicating that high methyl esterified pectins facilitate the cell wall loosening. The de-esterification of the methylated pectins in the primary wall through pectin methyl esterase (PME) activity has been reported to be a characteristic feature in cambial derivatives towards xylem, which allows its greater radial growth [27]. However, reappearance of less dense, electron-opaque patches in the intercellular spaces in the cell corner region after initiation of secondary wall deposition indicates the reappearance of pectins in this region. Our experiment with JIM5 labelling shows strong labelling during the reorganisation of cell corner middle lamellae after secondary wall deposition, suggesting that the formation of egg box by Ca^2+^ mediated ionic cross-linking of low methyl esterified HG [28] occurs during advanced stages of fibre development in *L. leucocephala*.

Immunodetection of β(1–4)-galactan (LM5) revealed a strong labelling from the cell walls of cambial zone cells in *L. leucocephala*. The primary walls and middle lamellae of cambial cells of conifers are reported to contain a high proportion of pectic polysaccharides, including homogalacturonan and RG1 with side chains of β(1–4)-galactan [29]. The absence of LM 5 labelling from middle lamellae of cells undergoing expansion and intense fluorescence after cell expansion also suggests β(1–4)-galactan could be one of the wall polymers closely associated with pectic polysaccharides. This observation also confirms the histochemical changes in pectins in middle lamellae of cells in the expansion zone, contrasted with ruthenium red staining. Another conspicuous feature found in the *L. leucocephala* fibres during the late stage of secondary wall deposition is labelling of β(1–4)-galactan during the disappearance of intercellular spaces. LM5 labelling is reported to be present only until the secondary wall thickening in the developing xylem tissue of poplar [30]. LM5 labelling pattern also supports the light and TEM observation that the pectins are deposited in intercellular spaces, followed by lignification in cell corners. Previous studies using the immunolocalization technique also suggest a close relationship between galactan and increased lignification in softwood tracheids [29, 31, 32]. Mannans are thought to be associated with cellulose in softwoods but are less abundant in hardwoods. Recent studies have also shown that lignification is controlled by other polysaccharides such as mannans and xylans [19, 33] through chemical bonding with lignin to form lignin-carbohydrate complexes [23, 34]. The two types of xylan conformation formed by a specific pattern of 4-O-methylglucuronic acid and O-acetyl decoration in the xylan backbone might influence the xylan-cellulose (major domains with even substitution pattern) and xylan-lignin (minor domain with odd substitution pattern) interaction [35–37]. The present study shows an association of a larger amount of β(1–4)-galactan in the middle lamellae and cell corner, where increased lignin deposition has also been noticed. On the other hand, the xylan-rich secondary wall shows relatively less lignin concentration. Hence, xylans in the secondary wall of fibres in *L. leucocephala* appear to have a relatively weak relationship with the degree of lignification, while β(1–4)-galactan are closely associated with initiation and extension of lignification as previously reported [32].

Xyloglucan is a major hemicellulose component in the primary wall of dicot cell walls. In this study, we used the CCRCM1 antibody to visualise the dynamic changes in the distribution pattern of fucosylated xyloglucan [38] in differentiating xylem fibres of *L. leucocephala*. Weak CCRCM1 labelling during cell wall loosening of young fibres, while the appearance of labelling in the cell corner and compound middle lamella of fibres during the advanced stage of secondary wall deposition suggests the fucosylated xyloglucan also undergoes dynamic changes during fibre cell wall development in *L. leucocephala*. Previous studies have also shown CCRCM1 labelling in the compound middle lamella of *Arabidopsis* xylem fibres [39]. Our results agree with a previous report on the disappearance of CCRC-M1 labelling before secondary wall deposition during tracheary element differentiation from *Zinnia* mesophyll cell culture, possibly due to hydrolytic degradation and modification of xyloglucan during a specific developmental stage [40]. The changes observed in the distribution pattern of fucosylated xyloglucan suggest their function as a tether that plays a major role in loosening and tightening of cellulose microfibrils [41] during expansion growth and maturation of xylem fibres in *L. leucocephala*.

LM10 and LM11 antibodies demonstrated different xylan labelling patterns in the wood cell wall depending on their degree of substitution [42, 43]. The present study shows that during the early stages of secondary wall deposition in fibres, both less substituted (ls ACG Xs) and highly substituted (hs ACG Xs) xylans are deposited simultaneously, initially at the cell corner region. These initial events are similar to previous reports on the general secondary wall deposition process in the cell wall of dead fibres [42–44]. The beginning of xylan distribution from the cell corner of the S_1_ layer in the fibre may represent one of the initial steps in general secondary wall deposition [44], and it may not have any relationship with the initiation of lignification in the secondary wall [43]. In the mature fibres of poplar, LM10 labelling showed stronger xylan (ls ACG Xs) localisation in the outer secondary wall than inner layer, while LM11 revealed uniform distribution of hs ACG Xs in the whole secondary wall, indicating heterogeneous composition of xylans in the fibre cell wall [43]. The living fibres in *L. leucocephala*, on the contrary, LM11 labelling was intense from the outer part of the secondary wall layer, especially in the corner region, while LM10 labelling was uniform throughout the secondary wall. This suggests that variation can exist in the distribution pattern of xylans in living xylem fibres. According to Awano et al. [45], this heterogeneity in xylan distribution could be associated with intussusceptional or appositional mode of deposition of less substituted and highly substituted xylans.

Xylans are also proposed to be associated with changes in microfibril orientation, both in polylamallate cell walls that have alternating layers and in more conventional secondary walls where microfibril orientation changes among the typical layers [46]. In conifer tracheids, the results from a combination of two techniques, immunolocalization of xylans and polarisation microscopy, revealed that xylans with low levels of substitution (LM10) have a clear association with changes from disordered to transverse orientation at the margin of S_1_ layer and at S_3_ layer [32]. In the living fibres of *L. leucocephala*, on the contrary, highly substituted xylans (LM11) show strong labelling at the S_1_ region, especially at cell corners of the secondary wall.

The lignification in fibres occurred after maturation of cell walls in vessels and paratracheal parenchyma cells. The G residue absorbs much more UV light than the S-lignin residue and appears more fluorescent [47]. The incorporation of G units into the middle lamella of fibres during early developmental stages has been reported in differentiating xylem of Poplar [44]. In *L. leucocephala*, a gradual disappearance of pectins from the cell corners and compound middle lamella of region due to the masking effect of lignin has been noticed in fibres during their early stages of lignification, indicating the interaction of early deposited lignin with pectins in the CML region. The high lignin autofluorescence found in the middle lamella region of fibres might be due to the initial deposition of highly condensed G lignin units. During xylem cell differentiation from cambium, vessel walls deposit secondary wall faster and incorporate more condensed G units, whereas the fibre wall shows a slow secondary wall deposition and incorporation of more S lignin units. On the other hand, the S_3_ layer of fibres shows maximum lignin concentration compared to a large part of the secondary wall, while vessel walls showed much uniformity in lignin distribution in the secondary wall. According to Terashiama [48], lignification of cell walls proceeds in three distinct stages during maturation and the kind of monolignol changes with the type and age of the cell. Vessel walls finish their lignification much earlier than other elements and incorporate more G units, while S lignin is deposited mainly in the middle and late stages of lignification, as evident from other axial elements. Thus, xylem fibres become rich in S lignin, which might be due to differences in timing for the synthesis of different monomers by the protoplast. In Oak wood xylem elements, the period of lignification is thought to be one of the major factors affecting the heterogeneous distribution of G and S lignins [49]. Our study shows that the fibre wall is characterised by more S lignin units, while vessels, associated parenchyma and rays have more G units. These results are in agreement with the general concept of lignification pattern in which G units are initially incorporated, followed by S units [50]. Therefore, the observed similarity in heterogeneity in lignin distribution in living fibres of *L. leucocephala* and previous reports on dead xylem fibres suggests similar developmental changes in the incorporation of lignin monomers in both.

Transmission electron microscopy following ruthenium red staining of sections has shown the inhomogeneous distribution of pectic polysaccharides in the compound middle lamella region of fibres. Pectins are widely regarded as the intercellular glue, which, in addition to playing a role in cell adhesion [51] and in determining the porosity of the cell wall and therefore, has an important function in cell growth and differentiation [52]. However, the influence of pectin architecture on lignin distribution is far from clear. The examination of middle lamellae is more suitable for such studies, as it represents the common area where much of the pectin is located in the cell wall and lignification is initiated [53]. In the present study, the distribution pattern of pectins by ruthenium red and lignin by KMnO_4_ staining indicates inhomogeneous pectin distribution in the cell corner region during secondary wall deposition, which appears to have a correlation with corresponding inhomogeneity in lignin distribution. Based on an ultrastructural investigation on microdistribution of pectin, lignin and peroxidase in the Alfalfa xylem fibres, Wi et al. [53] reported that the distinct inhomogeneity in the distribution of pectin and lignin in the corresponding regions of middle lamellae suggests a possible relationship between these cell wall components. Apart from lignin inhomogeneity in cell corners, the presence of G units in the pectin-rich region also indicates the probable cross-linkage between these cell wall constituents. Therefore, our histochemical and ultrastructural results on pectins and lignin monomeric units in cell corners are in agreement with [54], who proposed that pectins interact with G units, whereas hemicelluloses cross-link with S units. In completely differentiated living fibres of *L. leucocephala*, the S_1_ layer showed less lignin deposition, while the transition zone of the S_1_ and S_2_ layers as well as the complete S_3_ layer is marked by an increased lignin concentration. Similar differences in lignin distribution pattern among secondary wall layers have also been observed in the fibres of Kenaf [55] and *Forsythia suspense* [5].

Delignification experiments showed the extraction of wall materials from the cell corner and compound middle lamellae regions while the primary wall remained intact. Similar results were observed in the fibres of *Fagus crenata* following delignification [45]. These authors reported that the simple method of delignification using sodium chlorite has no significant effect on the secondary wall, whereas xylanase treatment followed by delignification is more effective in delignification from primary and secondary wall regions. On the contrary, the present study demonstrates the removal of lignin from the secondary wall layers of *L. leucocephala* fibres without xylanase treatment. Our results agree with a previous report where a significant decrease in lignin concentration from the outer secondary wall layers of normal wood tracheids of *Cryptomeria japonica* was found following delignification using the sodium-chlorite method [29]. The observed difference in the effect of delignification might be related to interspecific variation in the proportion of cell wall chemical constituents.

## Conclusion

The present study reveals the developmental changes in the cell wall polymers and their distribution pattern during the differentiation of living fibres in *L. leucocephala*. The findings of the study could be useful in understanding the properties and specific utility of fibres as they are directly correlated with the cell wall structure and chemistry. The distribution pattern of JIM5, JIM7 and CCRCM1 labelling revealed the cell wall remodelling through dynamic changes in the molecular structure of homogalacturonan in terms of their degree of methylation and fucosylated xyloglucans for loosening and tightening of the cell wall during expansion and maturation stages of xylem fibre differentiation. The disorganisation and reorganisation pattern of β(1–4)-galactan, and pectins in the middle lamella region and intercellular spaces in the differentiating living fibres revealed by immunolabelling and ruthenium red staining, respectively, suggests pectins form the major cell wall polymer, and they may closely associate with intense lignification in these regions. The immunolocalisation of xylan showed a homogenous pattern of deposition during early stages of secondary wall formation. However, heterogeneity in the distribution pattern of highly substituted xylans (LM11) is noticed compared to the uniform distribution of less substituted xylans (LM10) in the secondary wall of mature fibres. The first deposited lignins in the compound middle lamella region are found to be guaiacyl units and copolymerisation of more syringyl units in the later stages. The ultrastructural studies indicate that the inhomogeneous lignin distribution in the cell corner middle lamella might be related to the corresponding inhomogeneity of pectins in this region. The delignification experiment showed that mild alkali treatment is effective in removing lignin from the secondary wall of wood fibres of *L. leucocephala*, indicating vulnerability of lignin in the fibre wall to mild concentration of chemical agents, which is a desirable feature from the perspective of the paper and pulp industry.

## Supplementary Information

Supplementary Material 1.

## Data Availability

Data sharing does not apply to this article as no datasets were generated or analysed during the current study.

## Abbreviations

SW: Secondary wall
CCML: Cell corner compound middle lamella
CML: Compound middle lamella
LR: London resin
G: Guaiacyl
S: Syringyl
TEM: Transmission electron microscopy
