# Supplementary material for: Temporal and spatial distribution of pectin, β(1–4)-galactan, xylan and lignin during differentiation of living fibres in young shoots of Leucaena leucocephala (Lam.) de wit
Source: BMC Plant Biol. 2025 Dec 1;25:1693. doi: 10.1186/s12870-025-07793-z (PMC12690861; doi:10.1186/s12870-025-07793-z)
Supplement: Supplementary file 1 — Supplementary Material 1. [file 12870_2025_7793_MOESM1_ESM.docx]

Supplimentary figures


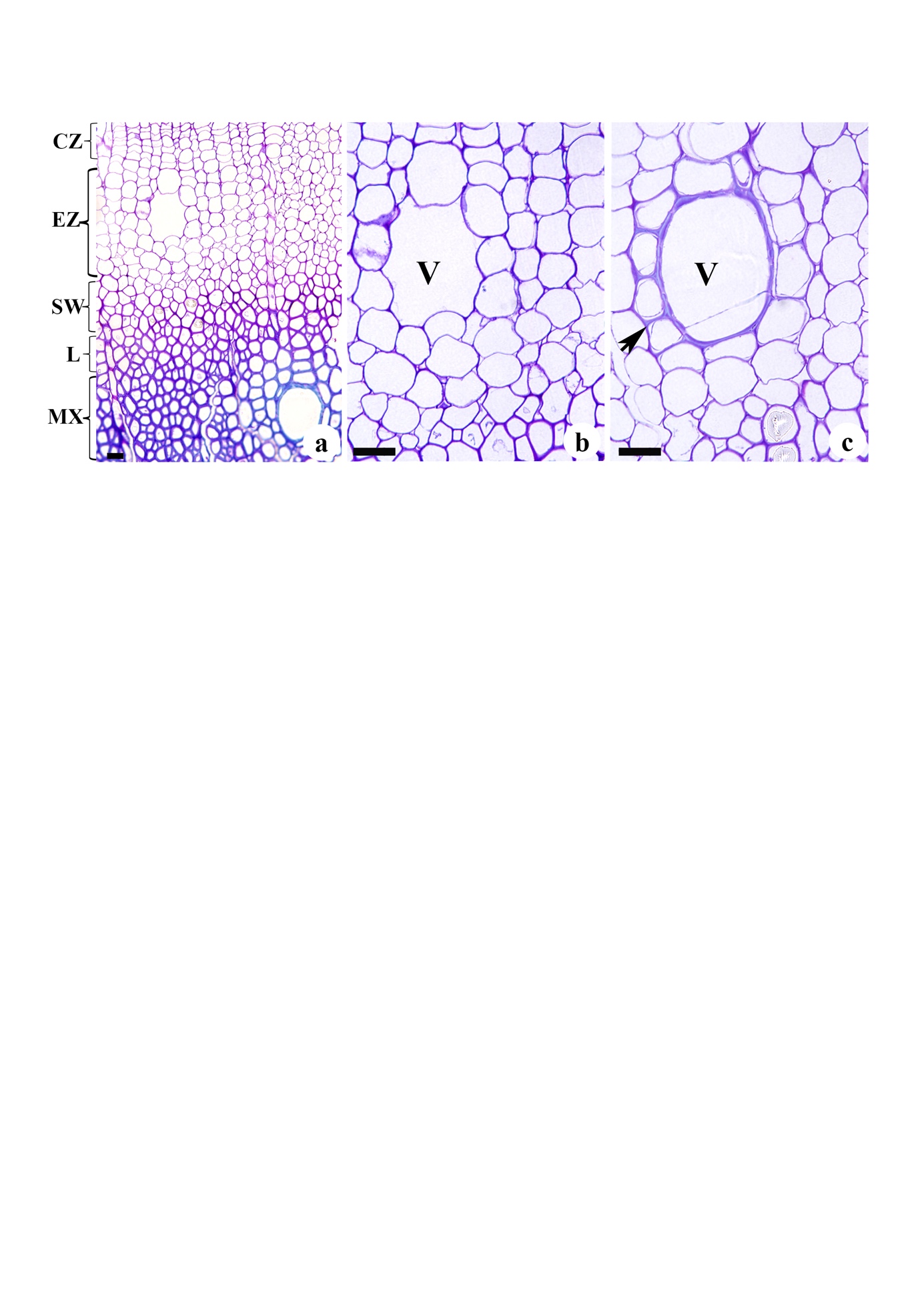


S1: (a) The developing xylem showing cells in the cambial zone (CZ) expansion zone (EZ), secondary wall deposition (SW), lignification (L) and mature xylem (MX). (b) Vessel (V) with primary wall near the cambial zone showing rapid expansion. (c) Vessel (V) and associated parenchyma cell showing secondary wall deposition and lignification earlier than other cell types. Scale bar=25 µm


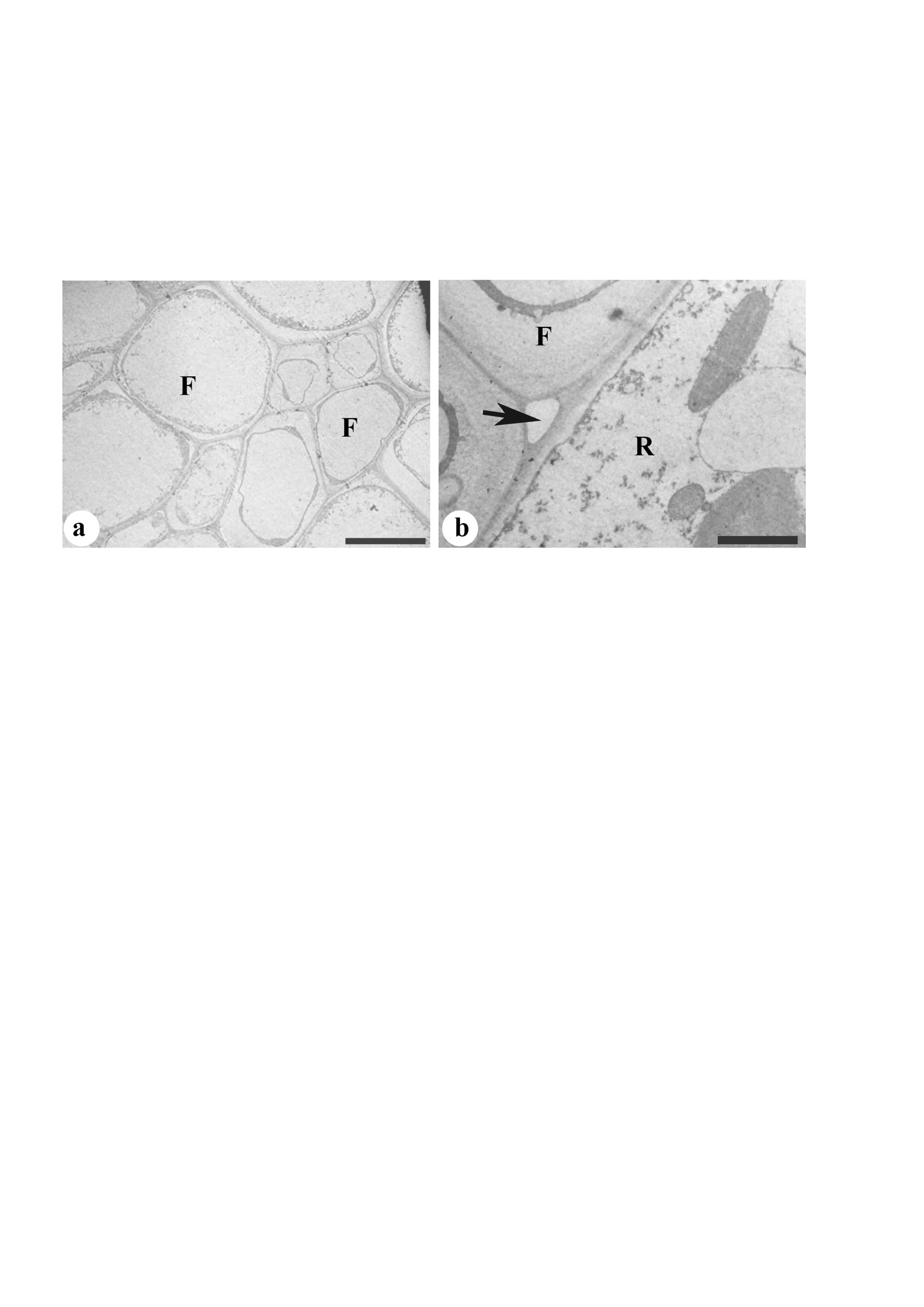


S2: (a) The developing fibres (F) in the expansion zone. (b) The fibres adjacent to ray (R) showing large electron translucent region in the CCML region (arrow). Scale bar a= 10 µm, b=2 µm


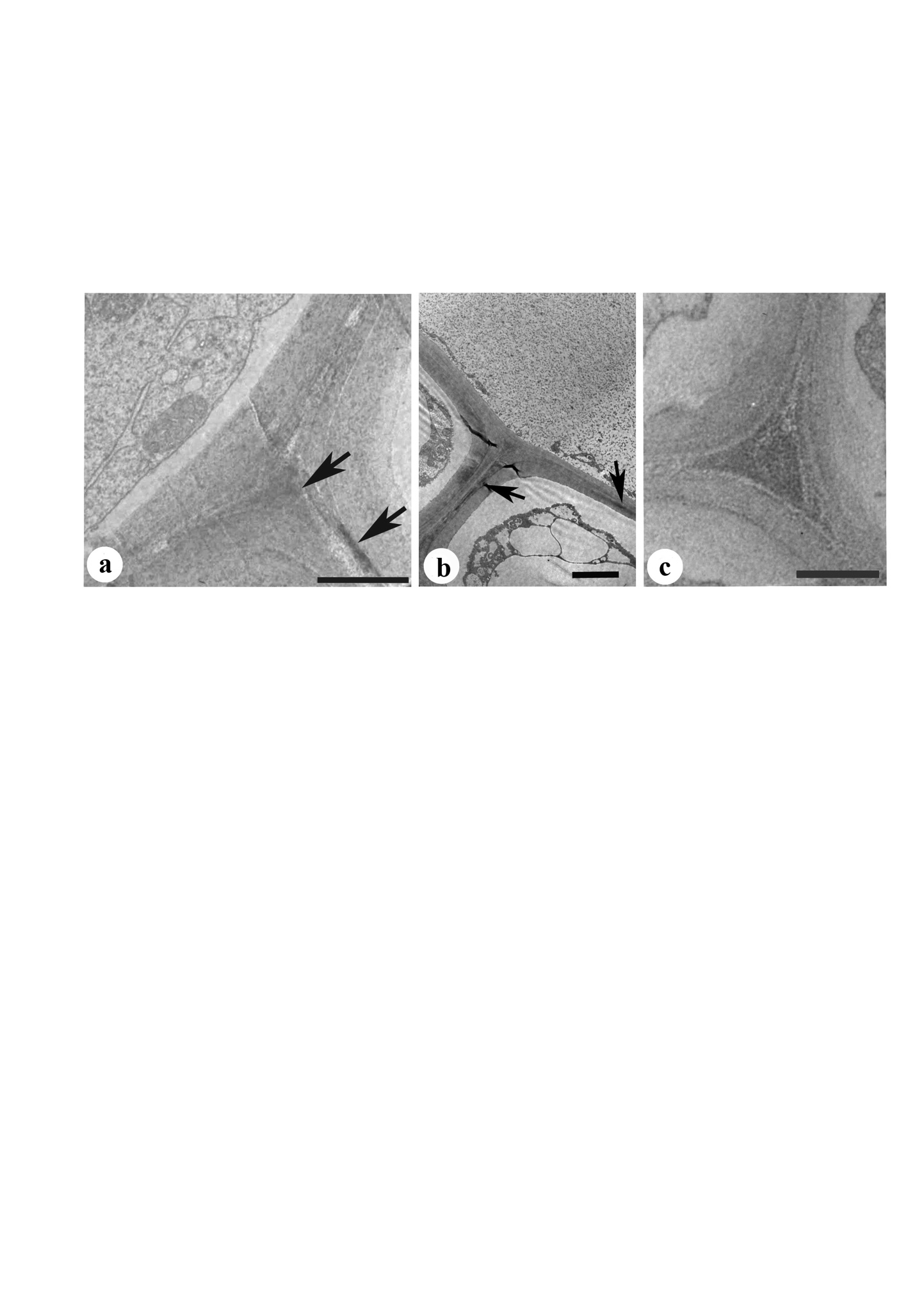


S3 (a-c): Fibres showing early stages of lignification in the cell corner and compound middle lamellae region (arrows). Scale bar= 1 µm.


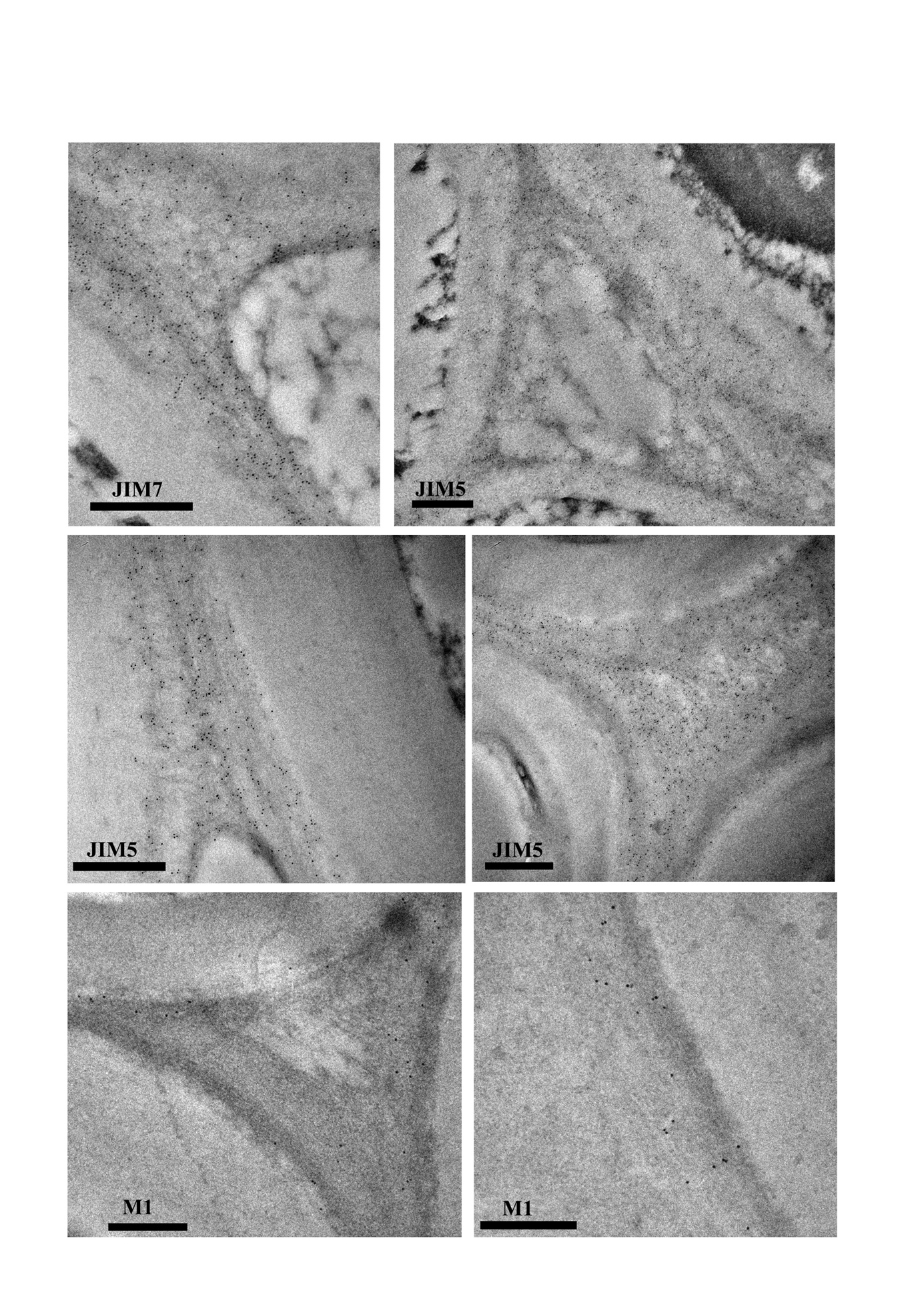


S4: Low magnified images of fibre area used in Figure 2 for immunolocalization of high methyl esterified homogalacturonans with JIM7, low methyl esterified homogalacturonans with JIM5, and fucosylated xyloglucans with CCRCM1 designated as M1. Scale bar= 1 µm.


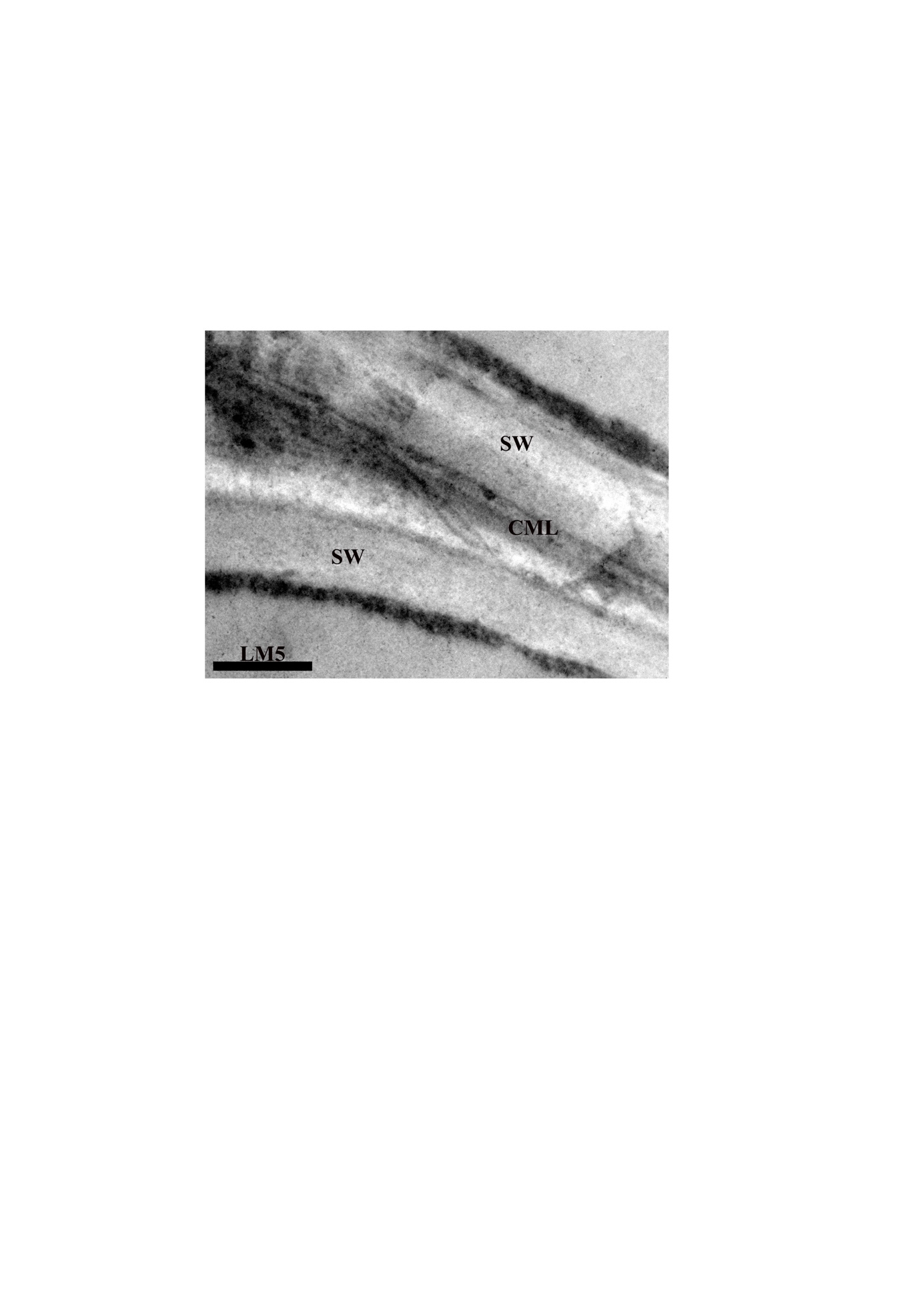


S5: Immunolocalization of β(1-4)-galactans with LM5 in the mature fibre showing weak labeling in both compound middle lamella (CML) and secondary wall (SW). Scale bar= 1 µm.
